# Bael (*Aegle marmelos*) fruit-based effervescent tablet formulations: Impact on physicochemical properties, bioactive compounds, and sensory attributes

**DOI:** 10.1016/j.heliyon.2024.e40544

**Published:** 2024-11-23

**Authors:** Md Rakibul Islam, S.M. Kamrul Hasan

**Affiliations:** Department of Food Processing and Preservation, Hajee Mohammad Danesh Science and Technology University (HSTU), Dinajpur, 5200, Bangladesh

**Keywords:** Bael fruit, Effervescent tablets, Physicochemical properties, Bioactive compounds

## Abstract

This study investigates the formulation and optimization of effervescent tablets made from freeze-dried bael (*Aegle marmelos*) fruit pulp, focusing on selecting appropriate excipients to enhance stability and ensure the effective release of its bioactive compounds for health benefits. The formulations—S_0_ (100 % fruit pulp), S_1_ (20 % citric acid), S_2_ (10 % citric acid and 10 % ascorbic acid), and S_3_ (20 % ascorbic acid) combined with equal parts of dried bael pulp, sodium bicarbonate, sugar, polyethylene glycol, and stevia were assessed for their physicochemical properties, bioactive compounds, and sensory study. The S_1_ demonstrated the fastest dissolution time (189 s), along with the lowest bulk density (0.488 ± 0.001 g/mL), tapped density (0.525 ± 0.001 g/mL), Hausner ratio (1.104 ± 0.114), and Cohesiveness index (0.075 ± 0.002), indicating better physicochemical properties. Among the formulations, the S_3_ showed the highest vitamin C (788 ± 0.05 μM AAE/g DM), total phenolic content (1289.17 μg GAE/g of DM), and total carotenoid content (19.3 ± 1.37 μM β-carotene E/g DM). The antioxidant and antidiabetic activity ranked: S_2_ ≈ S_3_>S_1_>S_0_. The presence of polyphenolic compounds in the bael fruit pulp was confirmed by high-performance liquid chromatography (HPLC) analysis. According to the sensory study, S_1_ stands out for its superior color, flavor, and satisfactory overall sensory experience; it exhibited a strong positive correlation with PC1, and highlights the critical sensory attributes influencing consumer perception. Therefore, these findings suggest that bael fruit-based effervescent tablets offer promising potential as a ready-to-drink product with beneficial health properties.

## Highlights

• Bael fruit pulp used as base material for effervescent tablet formulation.
• HPLC analysis confirmed nutritional and phytochemical existence in pulp powder.
• Effervescent tablets showed high bioactive and functional properties.
• Effervescent tablets showed strong sensory acceptance and promising properties.

## Graphical abstract

**Figure:**
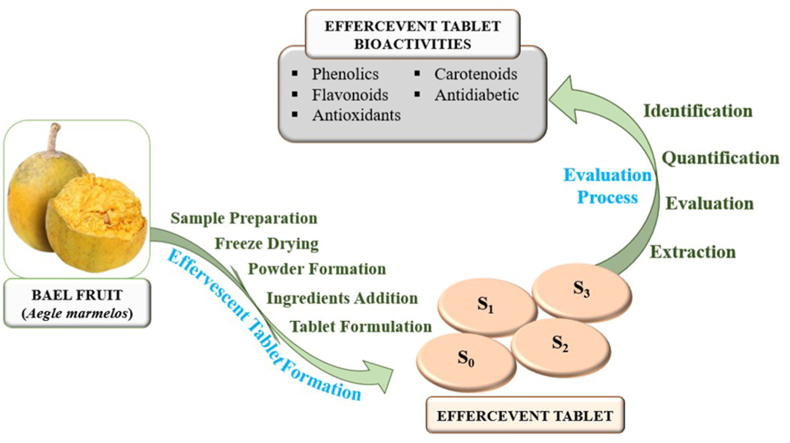

## 1 Introduction

In the realm of functional foods and innovative formulations, the exploration of natural sources for health-promoting products has gained considerable momentum [1]. Among the plethora of exotic fruits, the bael fruit (*Aegle marmelos*) stands out as a treasure trove of bioactive compounds with diverse physiological benefits [2]. Traditionally revered for its medicinal properties in various cultures, the bael fruit has recently garnered attention for its potential incorporation into novel formulations, such as effervescent juice tablets.

Effervescent formulations have gained popularity owing to their convenience, palatability, and enhanced bioavailability [3,4]. These tablets offer a refreshing and enjoyable means of consuming fruit extracts and present an intriguing platform for preserving and delivering bioactive compounds [5]. Prior to exploring the formulation of the tablet, it is imperative to understand the rich phytochemical composition of the bael fruit. Scientific investigations have unveiled a myriad of bioactive compounds within the fruit, including alkaloids, flavonoids, phenolic compounds, and essential oils [2]. These compounds have been associated with diverse health benefits, ranging from antioxidant and anti-inflammatory properties to potential antimicrobial and anti-cancer effects [6,7]. The pharmacological potential of bael fruit has been a subject of research interest, with studies suggesting its efficacy in managing diabetes, gastrointestinal disorders, and cardiovascular conditions [8]. As researchers strive to harness the health-promoting attributes of natural sources, the bael fruit emerges as a compelling candidate for incorporation into functional formulations.

Effervescent formulations have evolved into an ingenious vehicle for delivering bioactive compounds with improved bioavailability and consumer appeal [9]. The effervescent reaction, triggered by the combination of citric acid and sodium bicarbonate upon tablet dissolution, not only imparts a fizzy sensation but also enhances the solubility and absorption of bioactive compounds [10]. Accordingly, Tabasi et al. [11] evaluated the physicochemical and antioxidants of spray-dried barberry juice powder (50, 60, and 70 %) based effervescent tablets. Pei et al. [12] studied on the mixed fruit effervescent tablets compressibility and dissolution characteristics made from freeze-dried guava and pitaya fruit powders. Another study aimed to evaluate the physicochemical properties and flow characteristics of commercial fruit powders—pitaya, pineapple, mango, and guava—and their impact on the dissolution behavior of tablets formulated with effervescent agents [13].

In the context of bael fruit, the effervescent tablet format holds the promise of preserving the integrity of its bioactive constituents while offering a convenient and enjoyable mode of consumption. The effervescent matrix may act as a protective shield against environmental factors that could degrade sensitive compounds, ensuring the delivery of a potent and bioavailable product to the consumer [14]. The journey from bael fruit to effervescent tablet involves a meticulous interplay of physicochemical parameters. Factors such as tablet hardness, disintegration time, and effervescence dynamics play a pivotal role in determining the tablet's overall quality and consumer experience [15,16]. Tailoring these parameters to optimize the tablet's physicochemical attributes requires a nuanced understanding of the unique characteristics of bael fruit extracts. Recent research has explored how different formulation variables affect the physicochemical properties of effervescent tablets made with bael. The selection of excipients, such as binders and disintegrants, has been explored to achieve the desired tablet characteristics [17]. Understanding the interplay between these variables is crucial for developing a robust formulation that ensures both the stability of bioactive compounds and the appealing sensory attributes of the effervescent tablet.

As we navigate the realms of functional foods and nutraceuticals, the bael fruit emerges as a promising candidate for innovative formulations. Effervescent juice tablets, with their ability to enhance bioavailability and provide an enjoyable consumption experience, serve as a captivating avenue for translating the bioactive potential of bael into tangible health benefits.

This article aims to unravel the physicochemical intricacies of effervescent tablets based on bael fruit, shedding light on their potential as a functional food product. By synthesizing knowledge from phytochemistry, formulation science, and health applications, this exploration seeks to inspire further research and development in the domain of natural, bioactive-rich formulations.

## 2 Materials and methods

### 2.1 Sample collection and preparation

Fresh and properly ripe bael fruits were sourced from the Bangladesh Agriculture Research Institute (BARI) in Joydebpur, Gazipur, Dhaka, Bangladesh. Subsequently, the fruits were meticulously washed with normal tap water, ensuring the removal of any impurities from the outer shell. Following the washing process, the bael fruits were gently rubbed by hand and pat-dried using a clean cloth. The prepared fruits were then carefully split with a holy hammer, and the pulp was extracted with a spoon. The collected pulp was placed in a clean bowl and thoroughly mashed manually to achieve a smooth, uniform consistency.

### 2.2 Sample drying and powder formation

The prepared pulp sample was transferred into the stainless trays of the freeze dryer (Biobase Vertical Freeze Dryer BK-FD12P, China) to form a layer with a thickness of 15 mm. Each tray contained approximately 450 g of raw pulp. Initially, the trays were placed in the freezing chamber at −50 °C for 8–10 h. Once proper freezing was achieved, the vacuum pressure was set to 10 Pa, and condensation occurred at −60 °C. The drying process was accomplished at 30 °C for 48 h. Following the drying phase, the sample was collected and ground with the help of an electric grinder (Japian, IS-4250, India) and screened with a mesh 30 to obtain a powder with a particle size of 0.20 mm. The powdered samples were subsequently stored at −18 °C for later analysis.

### 2.3 Preparation of beal effervescent tablets

The preparation of effervescent tablets from bael powder was conducted following the protocol of Tabasi et al. [11] and Saifullah et al. [13] with modifications. In detail, four formulations namely S_0_ (control), S_1_, S_2_, and S_3_, were created (Table 1), incorporating different percentages of effervescent agents while maintaining bael as the primary ingredient. A consistent amount of dried fruit pulp was utilized in each sample, except for S_0_, where 100 % dried bael powder was employed, serving as the control sample.

**Table 1:** Formulation of effervescent tablets from bael fruit pulp powder.

Materials (%)	Sample Treatments			
	S_0_	S_1_	S_2_	S_3_
Dried bael pulp	100	24	24	24
Citric acid	–	20	10	–
Ascorbic acid	–	–	10	20
Sodium bicarbonate	–	14	14	14
Sugar	–	39	39	39
Polyethylene glycol	–	2	2	2
Stevia	–	1	1	1

In the formulation, effervescent agents like citric acid and sodium-bi-carbonate were employed. A binding agent, namely polyethylene glycol, was utilized to provide a compact characterization of the formulated tablets, and sugar was added for sweetening purposes. After finalizing the four formulations, 3.5 g of the formulated powder was transferred into a tablet-making tool, resulting in tablets with a size of 25 mm in diameter and a thickness of 3 mm.

### 2.4 Physicochemical analysis of effervescent tablets

#### 2.4.1 Determination of moisture content

Moisture content (dry basis) was determined using a halogen moisture analyzer (XY-105MW, Shanghai, China). Following the instructions in the user manual, the wind cover, pan holder, tray holder, and sample tray were meticulously assembled and calibrated. Subsequently, a 2.5 g sample was placed in the tray and the analyzer was initiated. The moisture content reading was recorded upon completion of the analysis, which took a few minutes.

#### 2.4.2 Determination of pH and total soluble solids (TSS)

The pH and TSS of tablet samples were analyzed by dissolving a tablet into 200 mL of water using a digital pH meter and calibrated refractometer, respectively [3]. Then the pH and TSS values were recorded in triplicate for each sample.

#### 2.4.3 Tablet dissolving time

The dissolving time was measured by counting the total time required for a tablet to dissolve automatically about 200 mL of water at ambient temperature [3].

#### 2.4.4 Determination of bulk density and tapped density

The bulk density and tapped density of powder samples were determined according to the protocol described by Shadordizadeh et al. [18]. Firstly, 2 g of powder sample was taken into a measuring cylinder and was gently shaken to ensure the uniformity of the powder surface. The bulk density was calculated by determining the ratio of the sample weight to the volume of the sample. equation (1) used for this calculation is as follows:

(1) $$\text{Bulk}\;\text{density}\;\left(\rho_{b}\right)=\frac{\text{Mass}\;\left(m\right)}{\text{Volume}\;\left(V\right)}$$

After determining bulk density, the tapped density of the sample was assessed by manually tapping repeatedly until the powder volume changed in the cylinder. To end, equation (2) was used to calculate the tapped density as follows:

(2) $$\text{Tapped}\;\text{density}\;\left(\rho_{t}\right)=\frac{\text{Mass}\;\left(m\right)}{\text{Volume}\;\left(V\right)}$$

In both equations, m represents the mass of the powder (g), and V represents the volume occupied in the graduated cylinder (mL).

#### 2.4.5 Determination of powder cohesiveness and compressibility index

The Hausner Ratio (HR) is employed to assess the cohesiveness of the powder sample. According to the classification of cohesiveness based on HR, low cohesiveness is considered when the HR value exceeds 1.2, moderate cohesiveness is characterized by an HR value between 1.1 and 2.4, and a high level of cohesion is observed when HR is less than 1.4 [19]. equation (3) for calculating the Hausner Ratio (HR) is given by:

(3) $$\text{HR}=\frac{\text{Tapped}\;\text{density}\;\left(\text{TD}\right)}{\text{Bulk}\;\text{density}\;\left(\text{BD}\right)}$$

Additionally, the compressibility index (CI) can be performed using equation (4):

(4) $$\text{CI}=1-\frac{1}{\text{HR}}$$

### 2.5 Color analysis

The color characteristics of the effervescent fruit tablets were assessed using a digital colorimeter (BC-110/200, Biobase, China), focusing on parameters such as L∗ (lightness), a∗ (redness to bluishness), and b∗ (yellowness to greenness).

### 2.6 Determination of vitamin C

Vitamin C content was determined by the spectrophotometric method following the protocol of Rahman et al. [20] with slight modifications. In brief, 1 g of powder sample was taken in a clean falcon tube and 10 mL of 1 % metaphosphoric acid was added for extraction. After being stored in the dark at ambient temperature for 45 min, the mixture was filtrated with a vacuum filter using Whatman No. 4 filter paper. Then, 250 μL filtrate was taken in a 10 mL measuring cylinder, mixed with 2.75 mL 2,6-dichlorophenolindophenol, and kept in a dark for 25 min. Afterward, the absorbance was measured at 515 nm using a UV–Vis spectrophotometer (UV 1900i, Shimadzu. Japan), and the content of vitamin C was expressed as μM ascorbic acid equivalents per gram of dry matter (μM AAE/g DM).

### 2.7 Bioactive compounds analysis

#### 2.7.1 Extraction of bioactive compounds

The extraction of bioactive compounds from bael fruit-based effervescent tablets was conducted using the method outlined by Islam et al. [21]. Initially, 2.5 g of samples were dissolved in 50 mL of solvent (80 % methanol and 20 % distilled water) maintaining a ratio of 1:20 (w/v). The resulting mixture was subsequently placed on a magnetic stirrer set at speed-3 (100 rpm), with no specific temperature control, and the extraction process was carried out for 1 h. To obtain the extracts, the mixtures underwent centrifugation (MF 300, Hanil Science Industrial Co., Incheon, Korea) at 4000 rpm for 10 min. Following centrifugation, a 10 mL plastic syringe was used to transfer a portion of the supernatant and subsequently passed through a Whatman filter no. 1 before undergoing analysis.

#### 2.7.2 Determination of total phenolic content (TPC)

The TPC was determined according to Hasan et al. [22] with slide modifications. Briefly, 500 μL of sample extract was mixed with 500 μL Folin-ciocelteu solution in a 10 mL measuring cylinder. Then, 1 mL of 7.5 % NaHCO_3_ solution and distilled water (DW) was added to make the volume 10 mL. Then, the mixture was vortexed and kept in a dark place at room temperature for 35 min. After that, centrifuged the mixture at 4000 rpm for 10 min, and collected the supernatant. Finally, the absorbance was measured at 750 nm via UV–Vis spectrophotometer (UV 1900i, Shimadzu. Japan) using appropriate blanks for background subtraction. Standard gallic acid (0–20 μM) was used for the calibration curve and the results were expressed as μg gallic acid equivalent per gram of dry matter (μg GAE/g DM).

#### 2.7.3 Determination of total flavonoid content (TFC)

To determine the TFC of the samples, the colorimetric method was used as described by Islam et al. [23] with minor modifications. The TFC was calculated from a standard calibration curve for quercetin (0–300 μM) and expressed as μg quercetin equivalents per gram of dry matter (μg QE/g DM). Concisely, 1 mL of extract was dispersed in 4 mL of DW in a centrifuge tube and 0.3 mL of 5 % NaNO_2_ was added. After 5 min incubation, 0.3 mL of 10 % AlCl_3_ was introduced and allowed to react for 1 min. Subsequently, 2 mL of 1M NaOH and 2.4 mL of DW were added and the mixture was vortexed. The tubes were then centrifuged at 4000 rpm for 10 min and left in a dark place at room temperature for 15 min. Absorbance was measured at 510 nm, using a blank prepared in the same way by substituting methanol instead of the extract.

#### 2.7.4 Determination of DPPH free radical scavenging activity

The DPPH radical scavenging ability assay was performed using the protocol of Islam et al. [21]. A solution of 0.13 mM DPPH was made using 80 % methanol stirred in an ultrasonic cleaner for 15 min and kept in a dark place for 30 min. The absorbance of the solution was set between 0.650 and 0.80 at 515 nm using a spectrophotometer. Following this, 50 μL of the extracted sample was mixed with 1.950 mL of DPPH and vortexed. The mixture was then incubated in the dark for 30 min at room temperature. After incubation, the absorbance at 515 nm was recorded. The results were reported as μM Trolox equivalents per gram of dry matter (μM Trolox/g DM), determined from a Trolox standard curve.

#### 2.7.5 Ferric reducing antioxidant power (FRAP) assay

The FRAP assay was carried out according to the reference of Hasan et al. [24]. The FRAP reagent was prepared by mixing acetate buffer (pH 3.6), 20 mM iron (III) chloride solution, and 10 mM TPTZ solution (in 40 mM HCl) at the ratio of 10:1:1 (v/v), respectively. Firstly, 50 μL of the sample extract was taken in a falcon tube and 1950 μL of freshly prepared FRAP reagent was added. Then, the mixture was vortexed properly and incubated for 4 min. Subsequently, absorbance measurements were recorded at 593 nm, and the results were compared to a standard iron (II) sulfate solution. The values were reported as μM Fe(II) equivalent per gram of dry matter (μM Fe(II)E/g DM).

#### 2.7.6 Determination of total carotenoid content (TCC)

The TCC was measured by following the procedure of Hasan et al. [25] with some modifications. In brief, 1 g of the sample was taken in a conical flask, and 50 mL solution of n-hexene:acetone:ethanol at a ratio of 50:25:25 was added. Then, the mixture in the conical flask was covered with aluminum foil to avoid light and placed in an ultrasonic cleanser (Model: JP-010T, Skymen Cleaning Equipment Shenzhen Co. Ltd., China) for 10 min. After that, the mixture was centrifuged at 4000 rpm for 10 min. The supernatant was then collected, adjusted to a final volume of 50 mL with the extraction solvent, and stored in a dark environment. Finally, the absorbance was measured at 450 nm and the results were expressed as μM β-carotene equivalent per g of dry matter (μM β-carotene E/g DM), using β-carotene as the standard.

#### 2.7.7 Analysis of antidiabetic activity (ADA)

The α-glucosidase activity of effervescent fruit tablets was measured by the protocol of Hasan et al. [26]. In detail, 50 μL of the extracted sample was combined with 100 μL of o.1 U/mL α-glucosidase solution prepared in a 0.1 M phosphate buffer at pH 6.9. After allowing the reaction to proceed for 10 min at 25 °C, 50 μL of 5 mM PNPG solution in the same buffer was introduced and incubated at 25 °C for 5 min. The absorbance was subsequently recorded at 405 nm using a UV–Vis spectrophotometer. The results were expressed as μg acarbose equivalents per gram of dry matter (μg AE/g DM), with acarbose used as the standard.

### 2.8 HPLC analysis

The polyphenolic compounds in the bael fruit pulp were detected using HPLC-DAD, following the method designated by Ahmed et al. [27] with slight modifications. The analysis was conducted on a Shimadzu LC-20A HPLC system (Shimadzu, Japan) equipped with a binary solvent delivery pump (LC-20AT), an autosampler (SIL-20A HT), a column oven (CTO-20A), and a photodiode array detector (SPD-M20A), all controlled by LC Solution software. The chromatographic separation was achieved using a Luna C18 column (5 μm, 4.6 × 250 mm, Phenomenex, USA) maintained at 33 °C. The mobile phase consisted of solvent A (1 % acetic acid in acetonitrile) and solvent B (1 % acetic acid in water), applied in a gradient elution: 0.01–20 min (5–25 % A), 20–30 min (25–40 % A), 30–35 min (40–60 % A), 35–40 min (60–30 % A), 40–45 min (30–5% A), and 45–50 min (5 % A). The flow rate was set at 0.5 mL/min with an injection volume of 20 μL. Detection was carried out at a wavelength of 270 nm. The mobile phase was first filtered using a 0.45 μm Nylon 6,6 membrane filter and then degassed under vacuum before use. Standard stock solutions were created in methanol with phenolic compound concentrations varying from 4 to 50 μg/mL for calibration purposes.

### 2.9 Sensory analysis

The sensory properties of the effervescent tablets were evaluated through a quantitative description analysis (QDA) method. The panelists were thoroughly briefed on the study's objectives, sensory evaluation procedures, and ingredients used in the formulation to clarify the potential health consequences. The panelists were graduate students and teachers from food science and technology backgrounds. The panel comprised 15 judges, including 7 women and 8 men, with ages ranging from 25 to 55 years. The assessors have evaluated the four features of effervescent tablets for the sensory evaluation, which included color, flavor, taste, solubility, and overall acceptability based on a 9-point hedonic scale, where, 1 = dislike extremely and 9 = like extremely [24].

### 2.10 Statistical analysis

The experimental design employed a completely randomized approach with three replicates, and results are presented as mean values ± standard deviation (SD). Statistical analysis of the experimental data was conducted using SPSS software (IBM version 27) to perform variance analysis (ANOVA), with significance set at p < 0.05. Principal component analysis (PCA) was employed to evaluate the relationships between various tablet formulations and their sensory characteristics, while a heat map and dendrogram for bioactive compounds were prepared utilizing Origin software (Version 10.1.0.170, OriginLab, USA) [28].

## 3 Results and discussions

### 3.1 Physicochemical analysis of effervescent tablets

The study analyzed the physicochemical properties of effervescent tablets formulated with freeze-dried bael fruit powder across four different samples: S_0_ (control), S_1_, S_2_, and S_3_. The parameters assessed included moisture, pH, TSS, dissolution time, bulk density, tapped density, Hausner ratio (HR), and cohesiveness index (CI) (Table 2)**.**

**Table 2:** Physicochemical properties of effervescent tablets.

Parameters	Samples			
	S_0_	S_1_	S_2_	S_3_
Moisture (%)	8.88 ± 0.07^a^	8.82 ± 0.01^a^	7.62 ± 0.16^b^	5.31 ± 0.02^c^
pH	6.3 ± 0.10^a^	4.6 ± 0.20^c^	5.5 ± 0.10^b^	6.4 ± 0.10^a^
TSS (°Brix)	1.7 ± 0.02^a^	1.4 ± 0.01^c^	1.29 ± 0.01^d^	1.6 ± 0.10^b^
Dissolution time (s)	–	189 ± 21^a^	390 ± 35^b^	642 ± 50^c^
Bulk density (g/mL)	0.511 ± 0.001a	0.488 ± 0.001^c^	0.503 ± 0.007^b^	0.514 ± 0.002^a^
Tapped density (g/mL)	0.908 ± 0.001^a^	0.525 ± 0.001^b^	0.902 ± 0.010^a^	0.908 ± 0.001^a^
Hausner ratio	1.693 ± 0.075^a^	1.104 ± 0.114^b^	1.798 ± 0.033^a^	1.765 ± 0.013^a^
Cohesiveness index	0.440 ± 0.008^d^	0.075 ± 0.002^c^	0.453 ± 0.006^b^	0.760 ± 0.008^a^

*Values are mean ± standard deviation of three replicas. Different alphabet presented on each row indicate significant differences at* p *< 0.05.*

The moisture content of the effervescent tablets varied significantly (p < 0.05) across the samples, with the highest in S_0_ (8.88 ± 0.065 %) and the lowest in S_3_ (5.31 ± 0.017 %). The moisture content is a critical factor influencing the shelf-life and stability of effervescent tablets. The significantly lower moisture content in S_3_ suggests better storage stability, potentially due to the proportion of excipients used in the formulation. Lower moisture levels decrease powder adherence and enhance surface contact with water during rehydration [29]. In contrast, the higher moisture content in S_0_ may indicate less effective moisture barrier properties, which could lead to quicker degradation of the product. These findings are agreed with the formulations, the control sample (S_0_) contained only bael pulp powder without having any carrier or binding ingredients. An increase in carrier concentration leads to a decrease in the moisture content of black mulberry juice powder as reported by Fazaeli et al. [30].

The pH of the effervescent tablets ranged from 4.6 ± 0.2 (S_1_) to 6.4 ± 0.1 (S_3_), and showed rank: S_3_≈S_0_>S_2_>S_1_. The pH levels of the effervescent tablets are crucial in determining the solubility and release profile of the active ingredients [4]. The acidic pH of S_1_ could enhance the dissolution of the tablets, making them more effective as a rapid delivery system. The addition of ascorbic acid did not lower the pH of the sample. However, the near-neutral pH of S_3_ might be more suitable for consumers, who are sensitive to acidic formulations. The pH levels observed are consistent with the natural pH range of bael fruit, which is typically around 5–6 [31].

The TSS (°Brix) is an indicator of the soluble sugars and other soluble components present in effervescent tablet juice. The TSS values were highest in S_0_ and lowest in S_2_, with S_1_ and S_3_ showing intermediate values of 1.4 ± 0.01 and 1.6 ± 0.1, respectively. A lower TSS seen in S_2_ might contribute to a less sweet taste, which could be desirable or undesirable depending on consumer preferences [20]. The higher TSS in S_0_ indicates a greater concentration of soluble sugars, likely resulting in a sweeter taste and faster energy release upon consumption. Among the formulations, S_1_ appears optimal, as it demonstrates an intermediate TSS range.

**Fig. 1:**
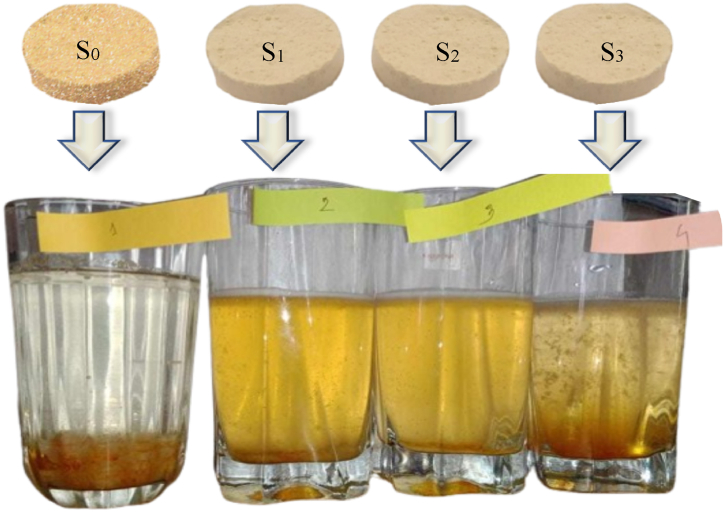
Dissolution of formulated effervescent tablets.

The dissolution times for the formulations varied significantly (p < 0.05), with S_1_ demonstrating the fastest dissolution at 189 s and S_3_ the slowest at 642 s (Fig. 1). Faster dissolution time is often desirable for effervescent tablets, ensuring quick release and action of active ingredients [15]. The prolonged dissolution time of S_3_ may be attributed to the nature of the excipients used. This is supported by the dissolution process, which involves a reaction between specific excipients – namely, an acid and an alkaline carbonate or bicarbonate – in an aquous environment, resulting in the production of carbon dioxide [32]. However, slower dissolution may also be beneficial in controlling the release of bioactive compounds, providing a sustained effect.

The bulk density ranged from 0.488 ± 0.001 (S_1_) to 0.514 ± 0.002 g/mL (S_3_). Tapped density showed less variation, with S_1_ having the lowest value (0.525 ± 0.001 g/mL) and S_0_, S_2_, and S_3_ sharing similar values around 0.908 g/mL. The bulk and tapped densities indicate the flow properties and compressibility of the powder mixtures. Lower densities in S_1_ suggest better flow properties, which could contribute to the faster dissolution observed. However, higher densities as seen in other formulations indicate better packing efficiency, potentially leading to slower dissolution but better tablet stability. Both bulk and tapped densities exhibited higher values than Naji-Tabasi et al. [28], who found 0.26–0.36 g/mL and 0.32–0.43 g/mL bulk and tapped densities of dried barberry paste powder, respectively. It was also clarified that higher drying temperatures resulted in increased tapped and bulk density values while drying time did not significantly affect tapped density (p < 0.05).

The Hausner ratio (HR) and Cohesiveness index (CI) are critical in understanding the flowability and compressibility of the tablet formulation. The HR was lowest in S_1_ (1.104 ± 0.114), indicating better flow properties, which is essential for consistent tablet formation and uniformity. These findings align with the observations made by Tabasi et al. [11], who noted that powders with an HR between 1.1 and 1.25 exhibit good flowability, while those with a ratio of 1.25–1.95 display poor flowability. In contrast, S_3_ exhibited the highest CI (0.760 ± 0.008), suggesting more cohesiveness, which could result in poor flowability and potential issues during tablet production. This high cohesiveness may also contribute to the longer dissolution time observed in S_3_ (Table 2). Overall, S_1_ emerges as the most promising formulation in terms of quick dissolution and ease of processing, while S_2_ stands out for its nutrient content.

### 3.2 Color analysis of effervescent tablets

**Fig. 2:**
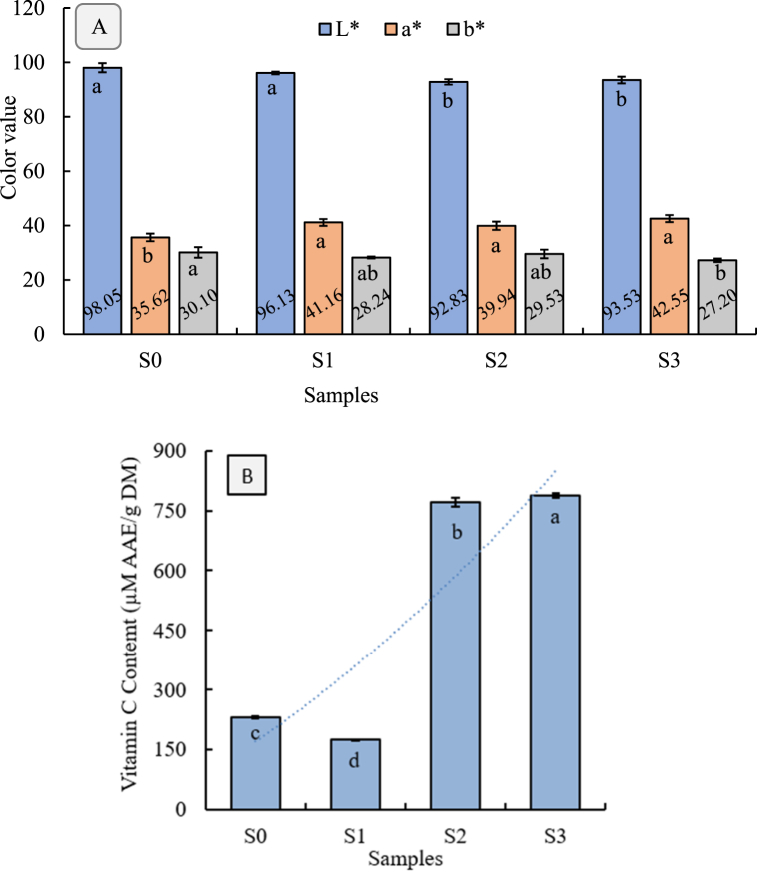
A) Color parameters and B) vitamin C content of bael pulp effervescent tablet prepared in different formulations. ^a-d^Different letters of each bar indicate significant differences between samples at p < 0.05.

Color is one of the key quality factors influencing consumers' rejection and acceptance of the final products [33]. In this study, the colorimetric properties (L∗, a∗, and b∗ values) of effervescent tablets were evaluated and presented in Fig. 2A. The lightness values of the samples demonstrated a notable variance (p < 0.05), with the control (S_0_) exhibiting the highest lightness (L∗ = 98.05 ± 1.67), indicating it is the lightest sample among others. In comparison, S_1_, S_2_, and S_3_ showed reduced lightness values of 96.12 ± 0.49, 92.82 ± 0.98, and 93.52 ± 1.26, respectively. The decrease in lightness in S_1_, S_2_, and S_3_ suggests the impact of the dried bael pulp on the overall color, potentially due to the inherent pigmentation of the bael fruit.

The a∗ values, which measure the red-to-green spectrum, were highest in S_3_, followed by S_1_ and S_2_, with the control (S_0_) having the lowest values. The increased a∗ values in the tablet formulations indicate a shift towards a redder hue, which can be attributed to the natural reddish pigments in the bael pulp. The presence of citric acid, ascorbic acid, and their mixture in the formulations resulted in the most substantial color change, aligning with findings that suggest phenolic degradation contributes to observed changes in color [34].

The b∗ values varied slightly among the samples. The control (S_0_) had a b∗ value of 30.10 ± 1.92, indicating a relatively yellowish tone. In contrast, S_1_ and S_2_ showed b∗ values of 28.24 ± 0.35, and 29.53 ± 1.58, respectively, while S_3_ had the lowest b∗ value (27.20 ± 0.65), suggesting a slight reduction in yellowness. This trend implies that the addition of ingredients might have contributed to a decrease in the yellow component of the tablet's coloration, potentially balancing out with other color properties imparted by the fruit. The color variations among the samples highlight the influence of dried bael pulp on the appearance of the effervescent tablets. The reduced lightness and increased red component in S_1_, S_2_, and S_3_, compared to the control (S_0_), reflect the pigments characteristic and homogeneously distributed bael pulp content with other ingredients. However, the color of effervescent tablets is influenced by natural pigments such as carotenoids and phenolic compounds, which interact with excipients, causing pH variations, that lead to color changes [35,36]. Additionally, the drying method can impact the final color by either preserving or degrading the natural pigments during processing.

### 3.3 Vitamin C content in the tablet

Vitamin C is essential in food products as it enhances the antioxidant properties and boosts the immune-supportive benefits, making the supplement more effective in promoting overall health [5]. Fig. 2B represents the vitamin C content of effervescent tablet samples including S_0_, S_1_, S_2_, and S_3_. The results exhibited a significant difference among the samples (p < 0.05). The highest and lowest vitamin C content was presented by S_3_ and S_0_ (control) samples, respectively. The overall trends for vitamin C content were as follows: S_3_ > S_2_ > S_0_ > S_1_. The highest vitamin C content (788.05 ± 5.91 μM AAE/g DM) in the S_3_ effervescent tablet may be due to the presence of high ascorbic acid in the formulation. Consequently, the above findings were supported by the second-highest vitamin C content (771.26 ± 10.61 μM AAE/g DM) in S_2_, which contained a similar amount of ascorbic acid in the formulation. This suggests that the combination of citric acid and ascorbic acid can synergistically enhance the stability and activity of vitamin C, as citric acid acts as a metal chelator, improving the antioxidant effect when used alongside ascorbic acid [37]. On the contrary, the reason for exhibiting the lowest value (176.14 ± 0.21 μM AAE/g DM) in the S_1_ sample may be the absence of ascorbic acid as well as dilution effect in the formulation. In the absence of additional stabilizing agents like ascorbic acid, citric acid might have a detrimental effect on the stability of vitamin C, leading to its reduction over time. However, the combination of citric acid with ascorbic acid can enhance the antioxidant effect, helping to maintain vitamin C stability and potency in formulations [37].

### 3.4 Bioactive compounds in juice tablet

#### 3.4.1 Total phenolic content (TPC)

**Fig. 3:**
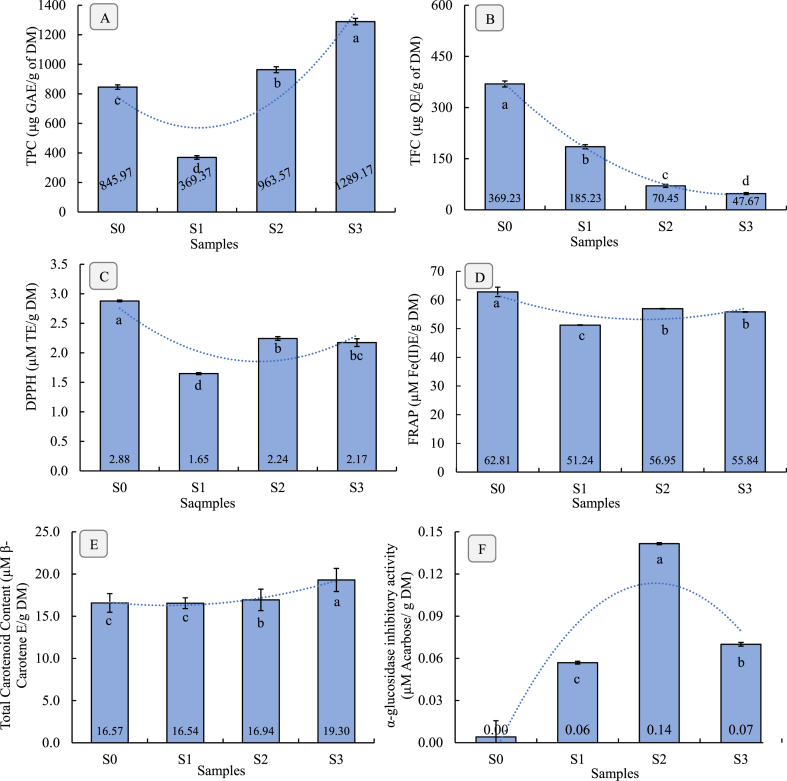
A) TPC, B) TFC, C) DPPH, D) FRAP, E) total carotenoid content, and F) α-glucosidase inhibitory activity of bael fruit effervescent tablets with different formulations. ^a-d^Different letters of each bar indicate significant differences between samples at p < 0.05.

The TPC of the effervescent tablets formulated from bael fruit pulp varied significantly across the different formulations and presented in Fig. 3A. The results indicated that S_3_ exhibited the highest TPC, whereas S_1_ showed the lowest values. The control sample (S_0_), which consisted solely of dried bael pulp, had a TPC of 845.97 mg GAE/g DM. The trends followed for higher TPC were as S_3_ > S_2_ > S_0_ > S_1_. As expected, the S_3_ contained the highest TPC value due to the presence of ascorbic acid (20 %) and the absence of citric acid in the formulation. Ascorbic acid exhibited high anti-oxidant properties, potentially stabilizing phenolic compounds during the formulation process [38]. According to Islam et al. [21], who clarified that TPC is highly correlated with antioxidant activity. Moreover, the lack of citric acid might have reduced the degradation of phenolics, as citric acid can sometimes cause phenolic degradation under certain conditions. The presence of sodium bicarbonate and sugar might also play a role in maintaining phenolic stability by creating a buffering environment and providing protection against oxidative degradation. The stability of phenolic compounds can be influenced by both storage conditions and the formulation ingredients. De Beer et al. [34] highlighted that the presence of different ingredients in formulations can impact phenolic stability, suggesting that certain components may either enhance or reduce degradation depending on their interactions.

The results indicate that the formulation components, especially the type and concentration of acids (citric acid and ascorbic acid), play a crucial role in determining the TPC of the final effervescent tablet. Ascorbic acid appears benefic ial in preserving or enhancing the phenolic content, while citric acid may contribute to phenolic degradation. These findings are consistent with existing literature, that highlights the role of acids in either stabilizing or degrading phenolic compounds during food processing [10].

#### 3.4.2 Total flavonoids content (TFC)

The TFC of effervescent fruit tablets showed significant (p < 0.05) variation across different formulations made from bael fruit pulp (Fig. 3B). The results indicate that the bael pulp is a rich source of flavonoids. Accordingly, the highest TFC was observed in the pure dried bael pulp (S_0_) with 369.22 μg QE/g of DM. However, during the formulation, the TFC was drastically reduced showing 185.22, 70.45, and 47.67 μg QE/g of DM for S_1_, S_2_, and S_3_, respectively. This reduction in TFC could be attributed to the dilution effect caused by the addition of other excipients such as citric acid, and ascorbic acid in the formulations. Moreover, the substantial decrease in TFC in S_1_, S_2_, and S_3_, compared to S_0_, indicates that the presence of ascorbic acid may influence the stability and retention of flavonoids during the formulation process, as the acids can sometimes degrade the polyphenols under certain conditions. Pérez-Ramírez et al. [39] noticed the degradation of some polyphenolic compounds such as sinapic acid, rutin, gallocatechin gallate, vanillin, and ellagic acid upon the addition of citric acid and stevia on roselle fruit beverage.

#### 3.4.3 DPPH radical scavenging activity

Antioxidants as either food additives or supplements derived from plant sources exhibited a safeguarding effect to minimize reactive oxygen species, and protect against cardiovascular and degenerative diseases [40]. The DPPH radical scavenging activity of effervescent tablets exhibited a significant difference among the samples (p < 0.05), and the results are presented in Fig. 3C. The sequence of higher to lower DPPH radical scavenging activity value (μM TE/g DM) was S_0_>S_2_> S_3_> S_1_. The control sample (S_0_) displayed significantly higher DPPH activity than other formulated samples (p < 0.05), which may be the reason for the higher antioxidant properties present in the bael fruit pulp [41]. On the contrary, the inclusion of excipients in formulated samples S_1_, S_2_, and S_3_ appears to reduce the DPPH values, with S_1_ showing the lowest activity (1.65 ± 0.017 μM TE/g DM). This reduction could be attributed to the dilution of bael pulp or the replacement of the bael pulp with the added excipients like citric acid, ascorbic acid, and sodium bicarbonate, stevia which might interfere with the DPPH radical scavenging capacity. Formulation S_2_, which includes both citric acid and ascorbic acid, shows a moderate DPPH value (2.24 ± 0.031 μM TE/g DM), while S_3_, containing a higher concentration of ascorbic acid without citric acid, has a slightly lower value (2.17 ± 0.066 μM TE/g DM). Hasan et al. [25] reported that flavonoids are powerful antioxidant compounds. However, this study noticed a lower amount of TFC (Fig. 3B), thus, resulting in a lower DPPH radical scavenging capacity among the formulations. However, the overall formulation and the specific combination of excipients play a crucial role in modulating the antioxidant activity. These results highlight the importance of carefully selecting excipients in effervescent tablet formulations to maintain or enhance the desired bioactive properties, particularly antioxidant activity.

#### 3.4.4 Ferric reducing antioxidant power (FRAP) assay

The FRAP assay results in the bar graph (Fig. 3D) demonstrate a significant (P < 0.05) variation in antioxidant power across different formulations of effervescent tablets prepared from bael fruit pulp. Specifically, sample S_0_, which consists solely of dried bael pulp powder, exhibited the highest antioxidant power at 62.81 ± 1.64 μM Fe (II) Equivalent/g DM. This suggests that adding other excipients, particularly citric acid, ascorbic acid, and sugars, as seen in S_1_, S_2_, and S_3_, may slightly reduce the antioxidant capacity of the formulations. Among these, S_2_, which included a balanced combination of citric acid, and ascorbic acid showed a moderate antioxidant activity of 56.95 ± 0.093 μM Fe (II)E/g DM, indicating that the presence of ascorbic acid might help to partially retain the antioxidant properties. These findings agree with Fernanda et al. [42], who found a strong correlation between FRAP and ascorbic acid content in effervescent powder from *Solanum betaceum* fruit. However, S_1_ exhibited the lowest FRAP value at 51.24 ± 0.078 μM Fe (II)E/g DM, possibly due to the absence of ascorbic acid, highlighting its crucial role in maintaining the antioxidant potential of bael fruit-based formulations. Antioxidant compounds can protect humans and other organisms by breaking down oxidative stress causing free-radical chain reactions employing donating hydrogen atoms [38]. Overall, these results underline the importance of excipient selection in preserving the antioxidant benefits of bael pulp in effervescent tablet formulations.

#### 3.4.5 Total carotenoid content (TCC) in tablets

The analysis of TCC in effervescent tablets formulated from bael fruit pulp reveals significant differences (p < 0.05) among the samples (Fig. 3E). The highest TCC was recorded in S_3_ (19.3 ± 1.37 μM β-carotene E/g DM), followed by S_2_ > S_0_ > S_1,_ with values of 16.93 ± 1.27 > 16.57 ± 1.10 > 16.54 ± 0.64 μM β-carotene E/g DM, respectively. The elevated TCC in S_3_ is likely attributed to the higher concentration of ascorbic acid, which may play a dual role in preserving carotenoids and enhancing their extraction efficiency. This findings aligns with the study by Choi et al. [43] which demonstrated that the addition of ascorbic acid in orange juice contributed to both color stability and enhanced carotenoid preservation. The results indicate that the formulation in S_3_ is superior in retaining or enhancing carotenoid content, which is crucial for the antioxidant potential of the product. This observation agreed with Hasan et al. [44], who claimed that carotenoids act as a source of antioxidants leading to the prevention of diseases and improvement in immune systems. Therefore, the findings in this study are supported by the formulations, highlighting the unique composition of S_3_ as a key factor in maximizing carotenoid content.

#### 3.4.6 Antidiabetic activity in tablets

The α-glucosidase inhibitors are commonly suggested as an effective method to manage type 2 diabetes by reducing glucose absorption into the bloodstream [33]. Synthetic α-glucosidase inhibitors like acarbose, miglitol, and voglibose are commonly used to manage blood sugar levels, but they often come with side effects such as weight gain, bloating, hypersensitivity, diarrhea, and potential toxicity [21]. As a result, there is growing interest in natural products with inherent α-glucosidase inhibitory properties due to their effectiveness, safety, and lower toxicity, making them a promising alternative for treating diabetes mellitus [45]. Accordingly, the α-glucosidase inhibitory activity of the effervescent tablets formulated with bael fruit pulp demonstrated significant variation across the different formulations, as illustrated in Fig. 3F. The S_2_ sample demonstrated the highest inhibitory activity at 0.14 ± 0.001 μM AE/g DM, indicating a modest enhancement in enzyme inhibition, likely due to the combination of ingredients. The S_1_ and S_3_ exhibited lower activities at 0.06 ± 0.001 μM AE/g DM and 0.07 ± 0.001 μM AE/g DM, respectively, suggesting that these formulations, despite containing similar components, were less effective in inhibiting α-glucosidase. The control sample, S_0_, showed minor inhibitory activity (0.0 ± 0.012 μM AE/g DM), confirming that the active inhibition is primarily attributable to the added excipients rather than the bael pulp alone. Moreover, the freeze-dried bael fruit pulp exhibited a higher α-amylase inhibitory activity (78.7 ± 0.71 %) in the antidiabetic activity assay described by Hazra et al. [6]. In another study, the results demonstrated that *Aegle marmelos* exhibited stronger inhibitory effects on α-amylase and α-glucosidase compared to acarbose, with IC_50_ values of 123.65 μg/mL and 141.56 μg/mL, respectively [46]. Therefore, these results imply that the specific combination and concentration of ingredients in S_2_ may play a crucial role in optimizing α-glucosidase inhibition, which could be beneficial for the development of anti-diabetic effervescent tablets.

### 3.5 HPLC chromatographic profiling of bael fruit pulp

**Fig. 4:**
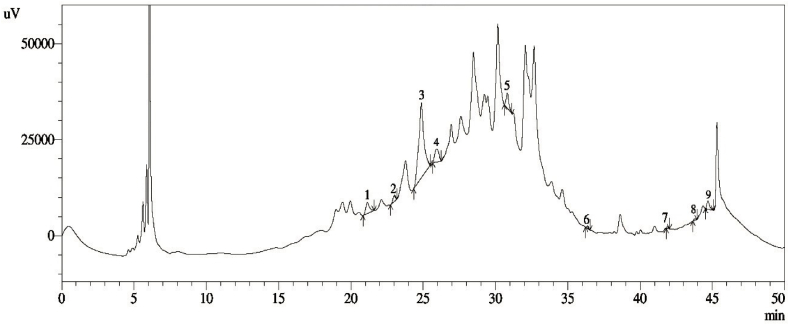
Typical HPLC chromatogram of 80 % methanolic extract of freeze-dried bael pulp: 1. Catechin hydrate, 2. Catechol, 3. (−) Epicatechin, 4. Syringic acid, 5. Rutin hydrate, 6. Rosmarinic acid, 7. Quercetin, 8. trans-cinnamic acid, and 9. Kaempferol.

The HPLC analysis of bael fruit pulp used to prepare effervescent juice tablets revealed a complex profile of constituents, with several distinct peaks indicating the presence of multiple compounds (Fig. 4). The chromatogram exhibited nine prominent peaks at retention times of approximately 21, 23, 25, 27, 30, 36, 42, 44, and 45 min, corresponding to the compounds catechin hydrate, catechol, (−) epicatechin, syringic acid, rutin hydrate, rosmarinic acid, quercetin, trans-Cinnamic acid, and kaempferol. These findings agreed with Chakrabarty et al. [47], who identified peaks of 16 polyphenolic compounds, among which 9 compounds matched the identified compounds with corresponding peak times. Another HPLC study conducted by Saha et al. [48] confirmed the presence of these compounds in bael fruit pulp by applying 17 different phenolic compounds as standards and identified eight major polyphenols: ascorbic acid, gallic acid, protocatechuic acid, p-coumaric acid, ferulic acid, quercetin, kaempferol, and apigenin.

The most prominent peaks were observed at 26 and 30 min, indicating that these are the major constituents of bael fruit pulp, such as syringic acid and rutin hydrate, respectively. Previous studies have identified rutin as a key flavonoid compound in bael fruit pulp [49], along with syringic acid [50]. The early eluting peaks at 21 and 23 min likely correspond to smaller or less polar compounds, whereas the later eluting peak suggests the presence of larger or more polar molecules. These findings indicate that the bael fruit pulp retains various bioactive compounds in the effervescent tablet form, which could contribute to its nutritional and therapeutic properties. These statements align with the information in a critical review on bael fruit's bioactive properties, medicinal values, and food applications [2]. The major compounds identified could be linked to the antioxidant, anti-inflammatory, and digestive health benefits traditionally associated with bael fruit [51]. According to Chakrabarty et al. [47], the flavonoid-O-diglycoside group includes compounds like quercetin, kaempferol, and myricetin, which are known for their anti-diabetic and anti-allergic properties. Further identification and quantification of these peaks using standard compounds could provide a deeper understanding of the specific bioactive compounds present and their respective concentrations in the effervescent tablet. This analysis underscores the potential of bael fruit pulp in developing functional food products with health-promoting benefits.

### 3.6 Heat map and hierarchical cluster analysis (HCA) of bioactive compounds in tablets

**Fig. 5:**
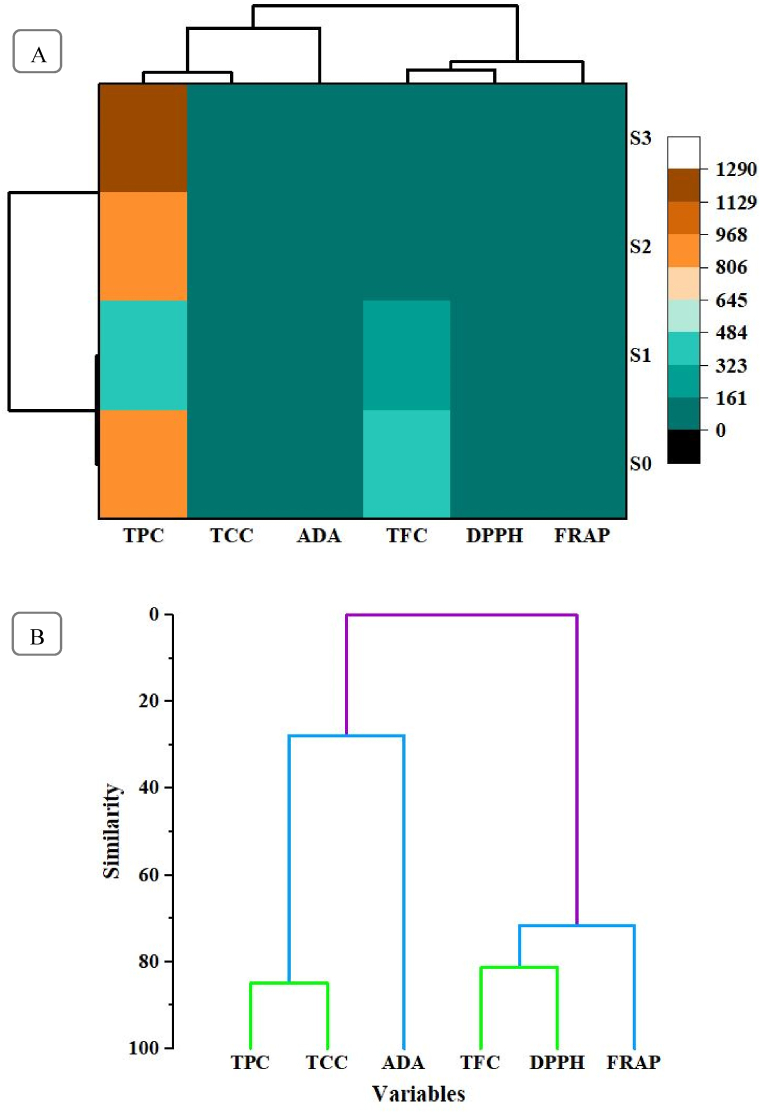
Heat map (A) and hierarchical cluster analysis (B) of bioactive compounds in bael fruit-based tablets.

The heat map graph (Fig. 5A) provides a visual representation of the distribution and intensity of bioactive compounds across different formulations of effervescent tablets using bael fruit pulp. This graph effectively highlights the variation in concentration levels of key phytochemicals such as antioxidants and anti-diabetic agents across the samples. The intensity of the colors in the heat map underscores the differences in bioactive compound profiles among the formulations, with certain samples showing higher concentrations, potentially indicating a more potent formulation. These insights are crucial for identifying the optimal formulation that maximizes the health benefits associated with bael fruit while maintaining desirable phytochemical properties.

HCA is a clustering technique that organizes samples into groups and subgroups, revealing a hierarchical structure. The outcome of HCA is typically displayed as a dendrogram, a tree-like diagram that illustrates the organization of samples and their relationships [28]. The dendrogram presented in Fig. 5B illustrates the hierarchical clustering of six variables such as TPC, TFC, DPPH, FRAP, TCC, and ADA. The analysis reveals two distinct clusters based on similarity. The first cluster groups TPC, TCC, and ADA, indicate a high degree of similarity among these variables, with TPC and TCC showing the closest relationship. This suggests that these three variables may share common pathways or be influenced by similar factors. The second cluster consists of TFC, DPPH, and FRAP, where DPPH and FRAP exhibit a particularly strong similarity, likely due to their shared involvement in antioxidant activity. TFC, while part of this cluster, shows a lower similarity, possibly due to differing mechanisms of action or varying contributions to the overall antioxidant potential. Similar findings were observed by Lourenço [52], who noted a cluster indicating that total antioxidant activity was similar only to TFC. This clustering pattern highlights the distinct roles these bioactive compounds play in the formulation and their potential synergistic effects in contributing to the overall functionality of the effervescent tablets. Accordingly, Zielinski et al. [53] utilized HCA as an effective and rapid chemometric tool to differentiate between 19 Brazilian frozen fruit pulp samples based on parameters like TPC, TFC, and antioxidant activity.

### 3.7 Sensory evaluation and principal component analysis (PCA) in juice tablet

**Fig. 6:**
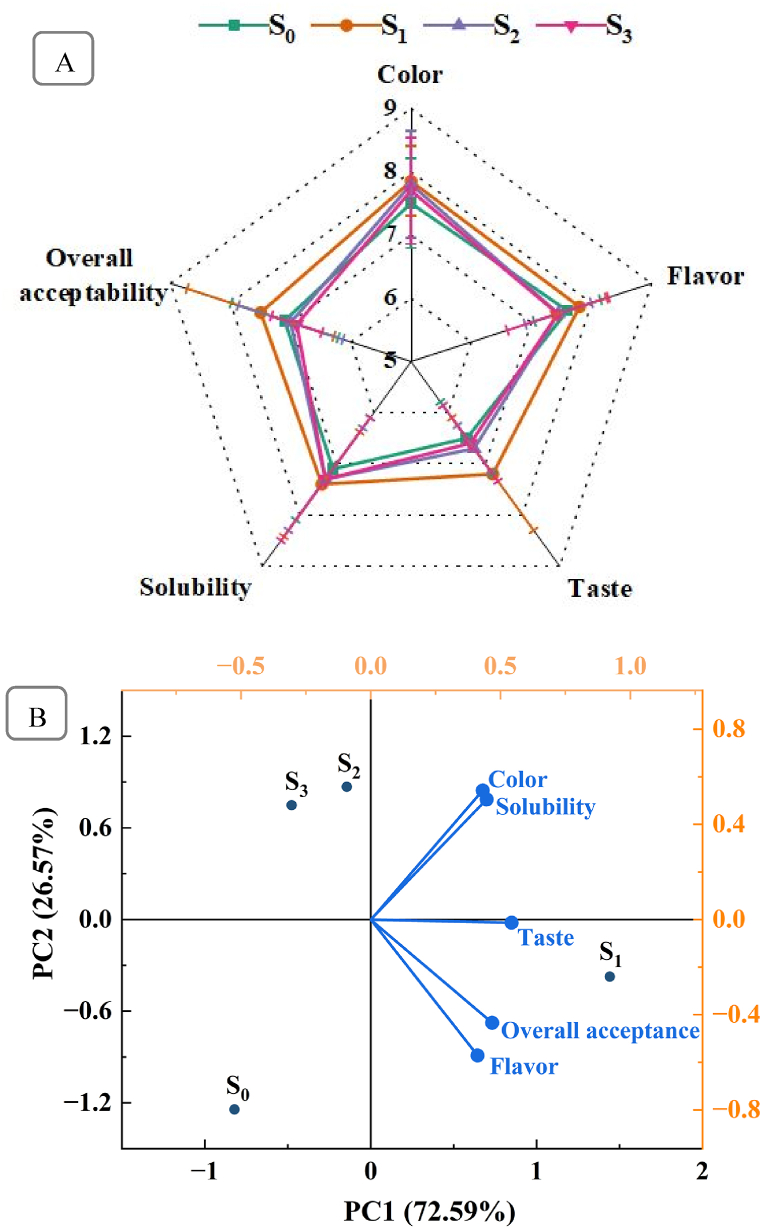
Sensory evaluation (A) and PCA analysis (B) of the effervescent tablet of bael fruit pulp with different formulations.

The sensory evaluation of effervescent juice tablets made from bael fruit pulp reveals distinct differences among the formulations (Fig. 6A). The visual character described by the color of the product “S_1_” secured the highest score (8) in hedonic rating followed by S_2_≈ S_3_ and S_0_. The reason for the high score in sample S_1_ might be the content of citric acid which preserves the color of the food products [54]. The flavor of the sample “S_1_” was moderately liked by the panelists and followed the rank: S_1_>S_0_>S_2_>S_3_. It can be claimed that the lower score in the samples S_2_ and S_3_ is due to ascorbic acid which gives a tart flavor. The hedonic rating on the taste and solubility of the sample were slightly different where the order of rank was: S_1_>S_2_≈S_3_≈S_0_. The sample S_0_ contains only pulp and, due to the absence of sugar and other ingredients, might have low sweetness and more sediment, thus, the panelists secured a lower score. Remarkably, the overall acceptability among the sample S_1_ was chosen by rating very much liking, and other samples (S_0_, S_2,_ and S_3_) were selected by rating moderately liking. These insights suggest that while S_1_ might attract initial consumer interest with its appealing color and flavor, it offers a more satisfactory overall sensory experience.

The PCA analysis of the effervescent tablets was carried out to investigate the predominant quality characteristics and to identify whether the tablet samples were different or similar concerning quality parameters [55]. The PCA graph effectively illustrates the relationships among sensory attributes of effervescent tablet samples (Fig. 6B). The first principal component (PC1) accounts for 72.59 % of the total variance, while the second principal component (PC2) explains 25.57 %. These two components jointly contributed 98.16 % of the grand gross variance, which was statistically satisfactory to differentiate all tablet samples [55]. Sensory attributes such as color, solubility, taste, flavor, and overall acceptability show strong positive correlations with PC1, indicating these attributes significantly dominated the tablet samples. Thus, suggesting all effervescent tablets are acceptable for consumption.

The tablet samples exhibit distinct profiles based on their positioning in the PCA plot. Sample S_1_, located positively along PC1 and closer to vectors for color, taste, flavor, solubility, and overall acceptability, indicates a high sensory score in these areas. Sample S_2_ and S_3_ are situated near the origin, suggesting an average performance across all sensory attributes with a positive association on PC2 and slightly negative on PC1, reflecting lower scores in most attributes and S_1_. Conversely, Sample S_0_, positioned negatively on both PC1 and PC2, demonstrates a unique sensory profile, distinct from other samples, with lower scores in color, taste, flavor, and overall acceptability. This comprehensive analysis highlights the critical sensory attributes influencing consumer perception and provides valuable insights for effervescent tablet development and quality improvement.

## 4 Conclusion

This study successfully developed and optimized effervescent tablets formulated with freeze-dried bael fruit pulp, demonstrating their potential as health-promoting, ready-to-drink products. Among the formulations, S_1_ stood out for its rapid dissolution time, ease of processing, and favorable sensory attributes, making it highly appealing for consumers. In contrast, S_2_ exhibited the strongest antioxidant and anti-diabetic activities, attributed to its rich bioactive profile, particularly its high levels of polyphenolic compounds. The presence of polyphenolic compounds in the bael fruit pulp, confirmed through HPLC analysis underscores the health benefits of these formulations. This suggests that both S_1_ and S_2_ are suitable candidates for commercial production, each offering unique advantages in terms of sensory appeal and health benefits. The presence of vitamin C, phenolics, and carotenoids further highlights the nutritional and functional potential of these formulations. Future research should focus on conducting long-term stability studies to assess the retention of bioactive compounds and overall effectiveness over extended storage periods. Additionally, exploring different ratios and combinations of excipients could optimize both the bioactivity and consumer acceptability of bael fruit-based effervescent tablets.

## CRediT authorship contribution statement

**Md Rakibul Islam:** Writing – original draft, Methodology, Formal analysis, Data curation. **S.M. Kamrul Hasan:** Writing – review & editing, Supervision, Project administration, Methodology, Data curation, Conceptualization.

## Data share statement

Data will be made available on request.

## Ethics statement

All relevant rules, guidelines, and regulations were followed, and consent was sought and obtained from all panelists/participants for sensory analysis in this study.

## Declaration of competing interest

The authors declare that they have no known competing financial interests or personal relationships that could have appeared to influence the work reported in this paper.
